# Assessing diet–microbiome associations with linear models: variability across human studies

**DOI:** 10.1080/19490976.2026.2740905

**Published:** 2026-10-05

**Authors:** Nicole M. Simm, Georgina M. Williams, Sophie Fowler, Katherine Barlow, Benazir Carter, Nicholas J. Talley, Simon Keely, Kerith Duncanson, Emily C. Hoedt

**Affiliations:** a School of Biomedical Sciences & Pharmacy, College of Health, Medicine and Wellbeing, University of Newcastle, Newcastle, NSW, Australia; b NHMRC Centre of Research Excellence in Transforming Gut Health, University of Newcastle, Newcastle, NSW, Australia; c Immune Health Research Program, Hunter Medical Research Institute, Newcastle, NSW, Australia; d School of Medicine & Public Health, College of Health, Medicine and Wellbeing, University of Newcastle, Newcastle, NSW, Australia; e Centre for Microbiome Research, Translational Research Institute, Queensland University of Technology (QUT), QLD, Woolloongabba, Australia; f School of Science, College of Engineering, Science and Environment, University of Newcastle, NSW, Newcastle, Australia

**Keywords:** Diet, gut microbiome, diet–microbiome associations, dietary assessment, microbiome methodology, methodological heterogeneity

## Abstract

Understanding and identifying relationships between dietary intake and the gastrointestinal microbiome can provide critical information for optimization of human health. However, methodological approaches largely differ and can impact research findings. Here we outline differences in reported diet–microbiome associations from a range of studies utilizing comparable statistical methods. From a subset of 25 studies using comparable methodology, only 3% of reported diet–microbe associations were common across more than one study. As such, we emphasize the inconsistencies of findings within the literature and wider implications of these differences.

## 1. Background

Research investigating how relationships between diet and the gastrointestinal (GI) microbiome influence human health is highly topical.^1^ Each year, thousands of papers report on diet–microbiome associations; however, inconsistencies in dietary assessment and analysis methods,^2^ microbiome sequencing,^3^ and statistical methods used in this area of research have revealed limitations in comparability and replication of findings.^4^ Here we report how inconsistent methodology may influence the accuracy of research findings and discuss the key limitations of current diet–microbiome research approaches.^5^

Our recent systematic scoping review examined and quantified methods used in diet and microbiome associative research and proposed guidelines for future practice.^6^ From this review, a subset of 25 studies using comparable high-quality approaches and statistical techniques were chosen to summarize and compare reported diet‒microbiome associations. Our comparison revealed inconsistent results across studies, with no more than two papers reporting the same microbial associations for each nutrient assessed. This led us to question the influence that research methodology has on subsequent findings in the diet‒microbiome research field.

## 2. Commentary

To investigate signal consensus in studies using comparable methodological approaches, our systematic scoping review^6^ included studies that reported nutrient composition analysis of whole dietary intakes in human populations and reported on associative microbiome analysis. In summary, we systematically searched for original research papers which reported metrics of both whole dietary intake (i.e., food frequency questionnaire, food recall, weighed food diaries) and the GI microbiome (using either 16S rRNA or metagenomic shotgun sequencing) and diet–microbiome associative analysis (i.e., Spearman, MaAsLin, LEfSe, linear regression).^6^ Our search identified 295 studies that captured either habitual or recent dietary intake and investigated associations with the GI microbiome. Of these studies, we screened for a subset of studies with comparable and high-quality methodological approaches that included analysis of nutrient composition, microbiome sequencing via Illumina platforms, and statistical methods of either linear regression, linear modeling methods, or MaAsLin2; a specialized implementation of multivariable linear modeling. We identified 25 studies (Table S1) that fitted the associative microbiome–diet analysis criterion which were further extracted to detail the associations between specific nutrient components and microbes at any taxonomic level (Table 1). Dietary collection tools included a range of validated food frequency questionnaires to reflect habitual intake, and both weighed food records and food recalls across 1–7 d to reflect recent intake (Table S1). While dietary collection tools varied substantially, nutrient components that were consistently defined across studies (i.e., protein, fat) were chosen for comparison. Directionality and effect sizes were not consistently available or extractable, so for the purposes of this work the associations were required to be significant in reporting from nominated *p*-values (e.g., q-value = 0.01 or *p*-value 0.05) and indicated as either positive or negative. Studies included a range of healthy and disease cohorts to explore where relationships between microbes and dietary substrates were dependent or independent of disease state.

**Table 1.** Summary of diet–microbiome associations in studies using linear modeling methods (*n* = 25), associations were required to be significant in reporting from nominated *p*-values and indicated as either positive or negative.

Taxon associated with:	Included studies							
FIBER (g/d)	Schoeps et al. [7]	Fuke et al. [8]	Le Roy et al. [9]	Kwon et al. [10]	Chansa et al. [11]	Jian et al. [12]	Zhang et al. [13]	Ferrocino et al. [14]
***Dietary timeframe:***	Habitual	Habitual	Habitual	Habitual	Recent	Recent	Recent	Recent
***Microbiome sequencing method:***	16S rRNA	16S rRNA	16S rRNA	16S rRNA	16S rRNA	16S rRNA	16S rRNA	16S rRNA
***Actinomyces***			**+**					
***Bacteroides***				**−**				
↳ *B. vulgatus* (ASV 353)	**−**							
↳ *B. eggerthii* (ASV 232)	**+**							
↳ *B. eggerthii* (ASV 319)	**+**							
↳ *B. uniformis* (ASV 20)	**+**							
***Bifidobacterium*** **sp. (ASV 41)**	**+**							
Blautia*			**+**			**−**		
						(−) Insoluble		
↳ *Blautia faecis* (ASV 30)	**+**							
***Butyricicoccus***				**+**				
**Christensenellaceae R7 group (ASV 481)**	**−**							
***Clostridium***						**+**		
↳ *C. sensu stricto 1* (ASV 120)	**+**							
↳ *C. sensu stricto 1* sp. (ASV 76)	**+**							
***Collinsella***						(+) Insoluble		
***Coprobacillus cateniformus*** **(ASV 578)**	**−**							
***Coprococcus 1***				**+**				
***Dialister*** **(ASV 135)**	**+**							
**Erysipelotrichaceae (ASV 461)**	**+**							
***Escherichia/Shigella***							**-**	
***Hydrogenoanaerobacterium***				**+**				
***Intestinibacter bartletti*** **(ASV 52)**	**+**							
***Lachnospira***			**−**					
***Lactococcus*** **sp**.**(ASV 385)**	**+**							
***Methanobrevibacter*** **(ASV 86)**	**−**							
***Negativibacillus***				**+**				
***Odoribacter***						**+**		
						(+) Soluble		
***Oscillospira***			**−**					
***Parabacteroides***				**−**				
↳ *P. distasonis* (ASV 209)	**+**							
***Proteobacteria***							**-**	
***Roseburia***								**+**
***Ruminoclostridium*** **5 (ASV 62)**	**+**							
**Ruminococcaceae**								
↳ NK4A214 group				**+**				
↳ unclassified (ASV 719)	**+**							
↳ UCG002					**+**			
↳ UCG004 (ASV 151)	**+**							
↳ unclassified (ASV 66)	**−**							
Ruminococcus*			**+**			**+**		
↳ *R. 1* (ASV 303)	**+**							
↳ *R. gnavus*			**+**					
↳ *R. torques group*					**+**			
***Streptococcus***		**−**						
		(−) Soluble						
		(−) Insoluble						
***Subdoligranulum***					**+**			
***Tyzzerella*** **(ASV 241)**	**-**							

CARBOHYDRATES (g/d)	Wang et al. [15]	Schoeps et al. [7]	Bolte et al. [16]	Qu et al. [17]	Chansa et al. [11]	Lapidot et al. [18]	Genoni et al. [19]
***Dietary timeframe:***	Habitual	Habitual	Habitual	Habitual	Recent	Recent	Recent
***Microbiome sequencing method:***	16S rRNA	16S rRNA	MGS	16S rRNA	16S rRNA	16S rRNA	16S rRNA
***Adlercreutzia equolifaciens*** **GCF 000478885**			**−**				
***Alistipes*** **(ASV 680)**		**−**					
***Bacteroides***							
↳ *B. uniformis*	**+**						
↳ *B. eggerthii* (ASV 319)		**−**					
↳ *B. intestinalis*				**−**			
***Barnesiella***						**−**	
***Bifidobacterium***			**+**				
↳ *B. adolescentis*			**+**				
↳ *B. adolescentis* strain unclassified			**+**				
↳ *B. longum*			**+**				
***Blautia***			**−**				
↳ *B. hydrogenotrophica* strain GCF 000157975			**−**				
***Catenibacterium***						**+**	
***Clostridium***						**−**	
↳ *C. sensu stricto*						**+**	
↳ *C. sensu stricto 1* sp. (ASV 76)		**−**					
***Dorea***			**−**				
↳ *D. formicigenerans*			**−**				
↳ *D. formicigenerans* strain unclassified			**−**				
↳ *D. longicatena* (ASV 22)		**+**					
***Eisenbergiella massiliensis*** **(ASV 579)**		**−**					
***Eubacterium hallii*** **strain GCF 000173976**			**−**				
***Faecalibacterium prausnitzii***			**−**				
***Fusicatenibacter***					**+**		
↳ unclassified (ASV 595)		**−**					
↳ *F. saccharivorans* (ASV 17)		**+**					
***Fusobacterium mortiferum***				**+**			
***Intestinibacter bartletti*** **(ASV 52)**		**−**					
***Lachnospira***						**+**	
**Lachnospiraceae**							
↳ bacterium 2 1 46FAA				**+**			
↳ unclassified (ASV 14)		**+**					
↳ unclassified (ASV 47)		**+**					
***Lactobacillus***							
↳ *L. muscosae*				**+**			
↳ *L. sakei*			**−**				
↳ *L. sakei* strain unclassified			**−**				
***Lactococcus***						**+**	
↳ *L.* sp. (ASV 385)		**−**					
***Methanobrevibacter*** **(ASV 86)**		**−**					
***Mogibacterium***						**+**	
***Oscillobacter*** **(ASV 251)**		**−**					
***Parabacteroides distasonis*** **(ASV 209)**		**−**					
***Roseburia***			**−**				**+**
↳ *R. inulinivorans* strain GCF 000174196			**−**				
***Ruminoclostridium*** **5 (ASV 62)**		**+**					
Ruminococcus*			**−**			**−**	
↳ *R. sp.* strain *1* (ASV 303)		**−**					
↳ *R. obeum*			**−**				
↳ *R. obeum* strain GCF 000153905			**−**				
↳ *R. obeum* strain unclassified			**−**				
↳ *R. torques*			**−**				
***Streptococcus***							
↳ *S. gordonii*				**+**			
↳ *S. mutans* strain unclassified			**+**				
***Turicibacter sanguinis*** **(ASV 74)**		**−**					
***Tyzzerella*** **(ASV 241)**		**−**					

FAT (g/d)	Wang et al. [15]	Schoeps et al. [7]	Fuke et al.[8]	Qin et al. [20]	Chansa et al. [11]	Lapidot et al. [18]	Ferrocino et al. [14]
***Dietary timeframe:***	Habitual	Habitual	Habitual	Habitual	Recent	Recent	Recent
***Microbiome sequencing method:***	16S rRNA	16S rRNA	16S rRNA	16S rRNA	16S rRNA	16S rRNA	16S rRNA
***Akkermansia***				**−**			
***Alistipes***							**+**
***Bacteroides***							
↳ *B. uniformis*	**+**						
↳ *B. eggerthii* (ASV 319)		**+**					
***Barnesiella***						**−**	
***Blautia*** **(ASV 496)**		**−**					
***Catenibacterium***						**+**	
***Clostridium sensu stricto***						**−**	
***Coprobacillus cateniformus*** **(ASV 578)**		**−**					
***Coprococcus 3 comes*** **(ASV 58)**		**+**					
***Eggerthella lenta*** **(ASV 191)**		**−**					
***Escherichia/Shigella*** **(ASV 9)**		**−**					
***Fusicatenibacter***					**+**		
***Lachnoclostridium***							
↳ unclassified (ASV 212)		**−**					
↳ unclassified (ASV 83)		**+**					
**Lachnospiraceae**							
↳ CAG 56 (ASV 143)		**+**					
↳ FCS020 group (ASV 380)		**+**					
↳ unclassified (ASV 14)		**+**					
↳ unclassified (ASV 47)		**+**					
***Lactococcus*** **sp. (ASV 385)**		**+**					
***Megamonas***			**−**				
***Mogibacterium***						**+**	
***Romboutsia*** **sp. (ASV 19)**		**+**					
***Roseburia***					**+**		
***Ruminoclostridium*** **5 (ASV 62)**		**+**					
**Ruminococcaceae**							
↳ UCG002					**−**		
↳ UCG014 (ASV 275)		**+**					
↳ unclassified (ASV 390)		**+**					
***Ruminococcus***						**−**	
↳ *R. 1* (ASV 303)		**+**					
***Streptococcus*** **(ASV 39)**		**−**					
***Subdoligranulum***							
↳ unclassified (ASV 306)		**+**					
↳ unclassified (ASV 8)		**+**					

MINERALS	Troci et al.[21]	Hammond et al. [22]	Wang et al. [15]	Fuke et al.[8]	Le Roy et al. [9]	Vinogradova et al. [23]	He et al. [24]
***Dietary timeframe:***	Habitual	Habitual	Habitual	Habitual	Habitual	Habitual	Recent
***Microbiome sequencing method:***	MGS	MGS	16S rRNA	16S rRNA	16S rRNA	16S rRNA	16S rRNA
***Alistipes A***	(+) Manganese						
***Bifidobacterium***						(−) Chloride	
↳ *B. adolescentis*		(+) Calcium					
***Clostridium asparagiforme***		(−) Calcium					
***Desulfobacterota***							(+) Phosphorus
***Enterococcus***						(−) Chloride	
***Escherichia***						(−) Sodium	
***Eubacterium***							
↳ *E. eligens*		(+) Calcium					
↳ *E. ventriosum*		(−) Calcium					
***Gemmiger*** **sp.**	(+) Manganese						
***Haemophilus parainfluenzae***		(+) Calcium					
***Lachnoclostridium***						(+) Chloride	
***Lachnospira***					(−) Magnesium		
***Ligilactobacillus***						(−) Chloride	
**Muribaculaceae UBA7173 sp.**	(+) Manganese						
***Olsenella***						(−) Chloride	
						(−) Sodium	
***Oscillospira***					(−) Magnesium		
**Oscillospiraceae CAG 103 sp.**	(+) Manganese						
**Proteobacteria**							(−) Copper
***Ruminococcus***					(+) Magnesium		
↳ *R. gnavus*					(+) Magnesium		
***Sellimonas intestinalis***		(−) Calcium					
***Streptococcus***				(−) Potassium			
***Sutterella stercoricanis***			(+) Ferritin				
			(+) Magnesium				

Fatty acids	Troci et al. [21]	Le Roy et al. [9]	Vinogradova et al. [23]	Lapidot et al. [18]	Wang et al. [25]	Hill et al. [26]
***Dietary timeframe:***	Habitual	Habitual	Habitual	Recent	Recent	Recent
***Microbiome sequencing method:***	MGS	16S rRNA	16S rRNA	16S rRNA	MGS	16S rRNA
***Allisonella***			(−) PUFA			
			(+) SFA			
***Anaerotruncus***					(+) TFA	
***Atopobium***				(+) MUFA		
				(+) PUFA		
***Bacteroides***				(−) PUFA		
				(−) SFA		
				(−) SFA		
***Barnesiella***				(+) PUFA		
***Catenibacterium***				(−) PUFA		
				(+) MUFA		
				(+) SFA		
***Clostridium sensu stricto***				(−) MUFA		
				(+) PUFA		
				(+) SFA		
***Dialister invisus***	(+) Octadeceno-tetranoic acid					
***Escherichia flexneri***	(+) Stearic acid					
***Klebsiella***			(+) SFA			
***Lachnobacterium***				(+) PUFA		
***Lachnospira***		(-) TFA		(+) MUFA		
***Lactococcus***				(+) PUFA		
***Megasphaera***			(+) SFA			
***Mogibacterium***				(−) SFA		
				(+) PUFA		
				(+) SFA		
***Odoribacter***				(−) PUFA		
***Oscillobacter*** **sp.**	(+) Tetra- cosanoic acid					
***Oscillospira***		(-) TFA				
**Oscillospiraceae**						
↳ ER4 sp.	(+) Linoleic acid					
↳ F23-Bo2 sp.	(-) Tetra- cosanoic acid					
***Paraprevotella***				(+) MUFA		
***Prevotella***					(−) TFA	
***Ruminococcus***			(−) SFA			
↳ R. gnavus*		(+) TFA				(−) TFA
						(−) Trans-octadecenoic acid
**Unclassified CAG273 sp.**	(+) Docosa- dienoic acid					
**Unclassified CAG417 sp.**	(+) Docosa- dienoic acid					

SUGARS	Egshatyan et al. [27]	Troci et al. [21]	Kwon et al. [10]	Chansa et al. [11]	Jones et al. [28]
***Dietary timeframe:***	Habitual	Habitual	Habitual	Recent	Recent
***Microbiome sequencing method:***	16S rRNA	MGS	16S rRNA	16S rRNA	16S rRNA
***Bifidobacterium***		(+) Galactose			
***Blautia***	(+) Starches				
***Butyricicoccus***	(−) Starches				
***Catenibacterium***			(−) Added sugar		
***Collinsella aerofaciens F***	(−) Sugar				
***Coprococcus 1***		(+) Glucose			
***Eubacterium***			(−) Added sugar		
↳ *E. eligens*					(−) Fructose
***Faecalibacterium***					
↳ *F. prausnitzii D*					(−) Fructose
↳ *F. prausnitzii F*		(+) Polypentose			
***Klebsiella***		(+) Polyuronic acid			
**Lachnospiraceae CAG 127 sp.**			(+) Added sugar		
**Muribaculaceae UBA7173 sp.**		(+) Oligo-saccharides			
***Prevotella*** **sp.**		(+) Oligo-saccharides			
***Romboutsia***		(+) Maltose			
***Streptococcus***			(−) Added sugar		
↳ *S. thermophilus*					(−) Fructose

ENERGY INTAKE (kJ or kcal/d)	Lopez-Montoya et al. [29]	Qin et al. [20]	Chansa et al. `[11]	Jian et al. [12]
***Dietary timeframe:***	Habitual	Habitual	Recent	Recent
***Microbiome sequencing method:***	16S rRNA	16S rRNA	16S rRNA	16S rRNA
***Akkermansia***		**−**		
***Bilophila***				**−**
**Erysipelotrichaceae unclassified genus**				**−**
***Fusicatenibacter***			**+**	
***Lactobacillus***	**+**			
***Parabacteroides***				**−**
**Peptostreptococcaceae unclassified genus**				**−**
**Ruminococcaceae unclassified genus**				**−**
***Ruminococcus***			**+**	

PROTEIN (g/d)	Schoeps et al. [7]	Bolte et al. [16]	Hasain et al. [30]	Lapidot et al. [18]
***Dietary timeframe:***	Habitual	Habitual	Recent	Recent
***Microbiome sequencing method:***	16S rRNA	MGS	16S rRNA	16S rRNA
***Agathobacter*** **(ASV 109)**	**+**			
***Bacteroides***				
↳ *B. plebeius* (ASV 253)	**+**			
↳ *B.* sp. (ASV 43)	**+**			
***Barnesiella***				**−**
***Bifidobacterium***		**−**		
↳ *B. adolescentis*		**−**		
↳ *B. adolescentis* strain unclassified		**−**		
***Blautia*** **(ASV 496)**	**−**			
↳ *B. faecis* (ASV 30)	**+**			
***Catenibacterium***				**+**
***Clostridium***				**−**
↳ *C. sensu stricto*				**+**
↳ *C. sensu stricto 1* sp. (ASV 76)	**+**			
***Coprobacillus cateniformus*** **(ASV 578)**	**−**			
***Coprococcus 3 comes*** **(ASV 58)**	**+**			
***Desulfovibrio***			**+**	
***Fusicatenibacter*** **(ASV 595)**	**−**			
**Lachnospiraceae**				
↳ CAG 56 (ASV 143)	**+**			
↳ unclassified (ASV 14)	**+**			
↳ unclassified (ASV 47)	**+**			
***Lactococcus***				**+**
***Leuconostoc***		**+**		
***Methanobrevibacter*** **(ASV 86)**	**−**			
***Mogibacterium***				**+**
***Parabacteroides distasonis*** **(ASV 210)**	**+**			
***Romboutsia*** **sp. (ASV 19)**	**+**			
***Ruminoclostridium*** **5 (ASV 62)**	**+**			
**Ruminococcaceae**				
↳ UCG014 (ASV 584)	**+**			
↳ unclassified (ASV 390)	**+**			
***Ruminococcus***				**−**
↳ *R. 1* (ASV 303)	**+**			
***Senegalimassilia*** **(ASV 627)**	**+**			
***Solobacterium moorei*** **(ASV 970)**	**−**			
***Streptococcus***				
↳ *S. australis* strain unclassified		**+**		
↳ *S. thermophilus* strain unclassified		**+**		
***Subdoligranulum***				
↳ unclassified (ASV 306)	**+**			
↳ unclassified (ASV 8)	**+**			

ALCOHOL	Egshatyan et al. [27]	Sakaguchi et al. [31]	Fuke et al. [8]	Bolte et al. [16]
***Dietary timeframe:***	Habitual	Habitual	Habitual	Habitual
***Microbiome sequencing method:***	16S rRNA	16S rRNA	16S rRNA	MGS
***Anaerostipes hadrus strain*** **GCF 000332875**				**+**
***Bifidobacterium***	**−**			
Blautia*		**+**		**+**
***Dorea formicigenerans***				**+**
↳ *D. formicigenerans strain unclassified*				**+**
***Eubacterium hallii*** **strain GCF 000173975**				**+**
***Faecalibacterium prausnitzii***				**+**
↳ *F. prausnitzii* strain unclassified				**+**
***Lactobacillus sakei***				**+**
↳ *L. sakei* strain unclassified				**+**
***Megamonas***			**+**	
***Prevotella***			**+**	
***Roseburia inulinivorans*** **strain GCF 000174195**				**+**
***Ruminococcus***				
↳ *R. obeum*				**+**
↳ *R. torques*				**+**
**Veillonellaceae**				**−**

CHOLESTEROL	Egshatyan et al. [27]	Vinogradova et al. [23]	Qin et al. [20]	Hasain et al. [30]
***Dietary timeframe:***	Habitual	Habitual	Habitual	Recent
***Microbiome sequencing method:***	16S rRNA	16S rRNA	16S rRNA	16S rRNA
***Actinomyces***		**−**		
***Akkermansia***		**+**	**−**	
***Bifidobacterium***	**−**			
***Catenibacterium***		**−**		
***Coprobacter***		**+**		
***Dorea***		**−**		
***Ligilactobacillus***		**−**		
***Roseburia***		**−**		
**Ruminococcaceae UCG002**				**−**
***Ruminococcus***		**−**		

VITAMINS	Troci et al. [21]	Fuke et al. [8]	Le Roy et al. [9]	He et al. [24]
***Dietary timeframe:***	Habitual	Habitual	Habitual	Recent
***Microbiome sequencing method:***	MGS	16S rRNA	16S rRNA	16S rRNA
***Bacteroides xylanisolven***	(+) Vitamin C			
***Coprococcus***			(−) Vitamin E	
***Desulfobacterota***				(+) Vitamin A
***Faecalibacterium prausnitzii F***	(+) Betacarotene			
	(+) Vitamin C			
**Lachnospiraceae TF01-11 sp.**	(+) Betacarotene			
***Megamonas***		(−) Vitamin B1		
***Oscillospira***			(−) Biotin	
			(−) Vitamin E	
***Prevotella***		(−) Vitamin B2		
***Ruminococcus***			(−) Biotin	

Note: **(+)** indicates a statistically significant positive correlation co-efficient and **(−)** indicates a statistically significant negative correlation co-efficient between diet-microbe. * indicates taxonomy that were reported as significantly associated with the same nutrient across > 1 study within this subset. Abbreviations: **ASV:** Amplicon sequence variants; **UCG:** Unclassified genera; **PUFA:** Poly-unsaturated fatty acids; **SFA**: Saturated fatty acids; **TFA**: Trans-fatty acids; **MUFA:** Mono-unsaturated fatty acids; **MGS:** Metagenomic shotgun sequencing. Bold bacteria names from Column 1 denote the highest level of taxon reported and related taxon clustered.

Overall, 233 microbe‒diet associations were reported with nutrients assessed using habitual food intake measures (e.g., food frequency questionnaires) and, 78 microbe‒diet associations were reported with nutrients assessed using measures for recent food intake (e.g., diet recall or record). Despite the large number of associations reported, 97% of reported associations were unique to only one study. Common taxon associations were found across four dietary variables: fiber, carbohydrates, alcohol, and trans-fatty acids. As species and strains of the same genus are likely to have the same function and dietary substrate preferences, collapsing associations to the genus-level increased overlap from 3% to 25%.

Signal consensus was seen with the genus *Ruminococcus* reported as positively associated with habitual fiber in a cohort of older adults from the general population^9^ and recent fiber intake in a cohort of middle-aged individuals with overweight or obesity.^12^ Positive associations with fiber were also seen with a range of *Ruminococcus* species, including an adolescent cohort with multiple sclerosis^7^ and middle-aged adults with overweight or obesity.^11^ ^,^ ^12^ Two studies reported an association between the genus *Blautia* and fiber intake, however, the direction of associations were opposite with a negative association with recent intake^12^ and positive association with habitual intake.^9^ The genus *Blautia* was positively associated with habitual alcohol intake in studies investigating a middle-aged cohort with IBD^16^ and an elderly cohort from the general population.^31^ These findings indicate that while some diet–microbiome associations are likely to be disease-specific, some are likely to be consistent across health and disease states. The only species-level association found consistent across studies was *Ruminococcus gnavus* with trans-fatty acids in older adults from the general population^12^ and middle-aged adults with overweight or obesity.^26^ Of the included papers, 52% reported microbiome data as deposited in an online repository, with the remaining stating that data were either available on request or not available.

## 3. Conclusion

Diet‒microbiome associations reported within associative studies are highly variable, even when comparable assessment and analysis methods are used. This variability is partially explainable by differences in population demographics and dietary intakes globally. The subset of 25 studies included were from a range of countries, age groups, and disease states (Table S1); all known variables that can impact on gut microbiota composition and the reporting of dietary intakes. However, we found some consistent dietary associations with *Blautia* and *Ruminococcus* taxonomy in both disease and general population cohorts across a range of age groups that suggest that some signals may remain consistent independent of health status. Beyond differences in cohort demographics, it is highly likely that differences in data collection, processing, and analysis are also major contributors to the disparity in findings. Methods used for collection of dietary intake and subsequent databases used for nutrient composition can vary significantly across demographics and countries, which could largely impact on study findings. Habitual methods of dietary intake such as food frequency questionnaires may not reflect daily short-term microbiome responses, whereas short-term collection methods may overlook how habitual dietary habits shape GI microbiome responses. Considering that dietary intake is a significant modifier of microbiome responses and dietary assessment tools vary greatly in terms of objectivity and specificity, consensus in dietary collection methods and collection timeframes is crucial to identifying signal consensus in future studies and should include both habitual and short-term measures of diet. Studies published with insufficient power for diet‒microbiome associative analysis may also limit the reproducibility and comparability of findings. Additionally, while the statistical methods in this subset of studies were similar in nature for handling diet‒microbiome associations (i.e., linear models/MaAsLin2), there were substantial differences in data preprocessing prior to modeling, such as multiple-testing corrections, confounder considerations, and transformation methods. These differences limited further consolidated analyzes. We noted that only a few papers described the statistical model parameters, covariates used, and statistical thresholds in detail, and this further highlights the immediate need to improve methodology reporting and pipeline harmonization to ensure comparability of future results. Data availability standards are largely improving across microbiome reporting studies; however, only half of this subset reported deposition of raw sequencing data, with no studies depositing raw dietary data for public access. This is a significant limitation that reduces the capacity for re-analysis or meta-analysis. As such, there is a need for unified approaches to study design, implementation, methods, and analysis of the associations within specific disease or condition subsets to progress understanding of diet‒microbiome associations. Minimum standards would include reporting metadata (inclusive of dietary variables), detailed statistical pipeline and preprocessing methods, and use of covariates in analysis. Improved availability of both raw microbiome data and dietary intakes will also assist in validation of methodological approaches and allow for cross-study comparisons.

## Supplementary Material

Supplementary Material Supplementary table 1.xlsx

## Data Availability

All data referenced in this letter were obtained from the published sources cited in Table 1. Data extraction was performed from associations reported in main texts, and supplementary data where available. As no re-analysis of raw data was conducted in this study, differences may exist in processing pipelines and covariate use in analysis that were not controlled for.
